# 6DoF Pose Estimation of Transparent Objects: Dataset and Method

**DOI:** 10.3390/s26030898

**Published:** 2026-01-29

**Authors:** Yunhe Wang, Ting Wu, Qin Zou

**Affiliations:** School of Computer Science, Wuhan University, Wuhan 430072, China; yunhwang@whu.edu.cn (Y.W.); wuting@whu.edu.cn (T.W.)

**Keywords:** robotic grasping, pose estimation, semantic segmentation, transparent object

## Abstract

6DoF pose estimation is one of the key technologies for robotic grasping. Due to the lack of texture, most existing 6DoF pose estimation methods perform poorly on transparent objects. In this work, a hierarchical feature fusion network, HFF6DoF, is proposed for 6DoF pose estimation of transparent objects. In HFF6DoF, appearance and geometry features are extracted from RGB-D images with a dual-branch network, and are hierarchically fused for information aggregation. A decoding module is introduced for semantic segmentation and keypoint vector-field prediction. Based on the results of semantic segmentation and keypoint prediction, 6DoF poses of transparent objects are calculated by using Random Sample Consensus (RANSAC) and Least-Squares Fitting. In addition, a new transparent-object 6DoF pose estimation dataset, TDoF20, is constructed, which consists of 61,886 pairs of RGB and depth images covering 20 types of objects. The experimental results show that the proposed HFF6DoF outperforms state-of-the-art approaches on the TDoF20 dataset by a large margin, achieving an average ADD of 50.5%.

## 1. Introduction

6DoF pose estimation aims to estimate the poses of objects, including 3D translation and 3D rotation. With the development of artificial intelligence technology, 6DoF pose estimation plays an important part in a wide range of applications, e.g., robotic grasping [1], industrial bin-picking [2], augmented reality [3], face alignment [4], and autonomous driving [5]. Although existing methods for 6DoF estimation have achieved remarkable success, 6DoF pose estimation of transparent objects remains a great challenge due to their transparency and lack of texture [6].

For non-transparent-object 6DoF pose estimation, several deep learning-based methods have been proposed in the past decade. PoseNet [7] pioneered the work in this field by introducing deep learning to 6DoF pose estimation. Subsequent studies have employed models such as PVNet [8], DenseFusion [9], and FFB6DoF [10], and have progressively improved accuracy through more reliable keypoint extraction or tighter RGB-D feature fusion. Holistic regression frameworks are generally more robust to truncation and occlusion [11], while DenseFusion demonstrated that integrating dense geometric and appearance cues can further boost robustness. PVN3D [12] and FFB6DoF unify pixel-wise keypoint voting with dense RGB-D fusion, bridging the gap between these research lines.

It is worth noting that FFB6DoF introduced a cascade feature fusion strategy, where RGB and depth features are fused at each convolution stage and passed to the next. The RGB sensor captures appearance information, while the depth sensor describes geometry. This cascade feature fusion strategy demonstrated better performance than the extract-then-fuse strategy of DenseFusion. However, RGB and depth belong to distinct domains. Fusing them at every stage can cause mutual interference, which weakens modality-specific feature extraction. This drawback becomes critical for transparent objects because their depth cues are already sparse [13].

In our work, we also investigate 6DoF pose estimation using keypoint prediction and hierarchical feature fusion. Specifically, we propose a hierarchical feature fusion strategy. Unlike the extract-then-fuse and cascade fusion mentioned above, our proposed strategy hierarchically fuses convolutional features. During the encoding process, features at different scales are fused hierarchically and concatenated to decoding layers. By fusing multiple feature maps at different scales, the geometric features of shallow layers and the semantic features of deep layers are fully utilized, improving the accuracy of 6DoF pose estimation for transparent objects. The main contributions of this work are two-fold:

- First, we propose a hierarchical feature fusion network, HFF6DoF, for 6DoF pose estimation of transparent objects. By hierarchically fusing the appearance information from RGB images and the geometry information from depth images, HFF6DoF achieves enhanced features and improved performance for 6DoF pose estimation on transparent objects.
- Second, we construct an RGB-D transparent-object 6DoF pose estimation dataset, TDoF20. This dataset consists of 31,108 single-object samples and 30,778 multi-object samples of 20 transparent objects captured in RGB-D images. Each image is annotated with 6DoF pose values, pixel-wise semantic masks, and restored depth images. This dataset will help promote further research in this field.

## 2. Related Work

In this section, we briefly review the 6DoF pose estimation methods for non-transparent and transparent objects.

### 2.1. 6DoF Pose Estimation of Non-Transparent Objects

Holistic methods directly estimate 3D object translation and rotation from images. Classic approaches relied on template matching to select the closest pre-rendered shape [14]. Deep learning methods attempt to regress 6DoF poses directly, either refining them iteratively [15] or discretizing the rotation space [16]. Others use flexible template sets, as well as training models with both rendered and real data [17,18]. The Model-Free Transformer Framework further explores texture-free pose estimation by leveraging geometry-based features from depth data, using transformer architectures to predict rotation and translation jointly without relying on CAD models [19].

Keypoint-based methods compute pose by detecting 2D or 3D keypoints. Typical deep learning-based methods estimate keypoints through heat maps [7,8] or regression [20,21]. PVN3D [12] introduces a 3D keypoint voting network to fully leverage point cloud features, achieving high performance on the LINEMOD [22] and YCB [23] datasets. Recently, CheckerPose [24] sampled a dense set of surface keypoints and refined 2D correspondences with a graph network, maintaining robust pose accuracy even under heavy occlusion. SD-Net [25] addresses symmetry-induced ambiguity in keypoint prediction through a robust 3D keypoint selection strategy and an effective filtering algorithm to eliminate multiple ambiguity and outlier candidates, achieving state-of-the-art performance on bin-picking scenarios. PoseFusion [26] employs a two-level nested U-shaped architecture with multi-scale keypoint correspondence to enhance keypoint refinement and establish accurate 3D-2D point correspondences for camera-to-robot pose calibration.

Dense methods predict 3D coordinates for each pixel and vote for the final result. Early work relied on random forests [27], while deep learning methods adopted CNNs for feature extraction and 3D coordinate prediction [28]. To enhance accuracy and robustness, some methods introduce joint classification and segmentation [29], disentangle rotation and translation [30], use discretized descriptors for dense surface representation [31], or output pose distributions [32].

Handling occlusion and symmetry remains a critical challenge in 6DoF pose estimation. Occlusion-Aware methods [33] leverage depth-guided graph neural networks to model non-adjacent relationships between surface points in occluded scenarios, adaptively fusing mask and binary code semantics to extract effective 2D-3D correspondences. For symmetric objects, PS6D [2] proposes symmetry-aware rotation loss and center-distance sensitive translation loss, using attention-guided multi-scale feature extraction and two-stage clustering to handle slender and multi-symmetric industrial workpieces in bin-picking applications.

Furthermore, PVNet [8], PVN3D [12], and FFB6DoF [10] combine keypoint-based and dense methods. They generate results for each pixel and obtain a set of keypoints through voting. These methods show high performance in the experiments, providing a valuable reference for 6DoF pose estimation of transparent objects.

### 2.2. 6DoF Pose Estimation of Transparent Objects

The reflective and refractive characteristics of transparent objects present significant challenges for detection, recognition, and 6DoF pose estimation. Early work combined edge distortion and specular cues to classify boundaries of transparent objects [34], but such features do not support reliable segmentation or 3D reconstruction [35]. With advances in hardware, some studies have adopted laser rangefinders [36], depth sensors [37], or structurally polarized light [38] to detect transparent objects. More recently, TA-Stereo [39] segmented transparent regions and homogenized their appearance so that stereo matchers could recover dense disparities over glass surfaces. FuseGrasp [40] extends the sensor repertoire by pairing a synthetic aperture mm-wave radar with the RGB-D input. Its two-stage network first completes the depth, and then refines grasp planning, which improves material recognition accuracy and grasp success.

The increasing adoption of RGB-D sensors has contributed to progress in 6DoF pose estimation for non-transparent objects, yet the depth signal is unreliable for transparent-object pose estimation. Traditional methods detected and recognized objects by extracting the local edges of transparent objects caused by specular reflection or refraction [37]. However, these methods generally lack accuracy under changeable lighting conditions. Some deep learning-based methods directly predict the bounding box of transparent objects [41], or utilize the characteristic of missing depth in transparent objects to locate objects and estimate poses [42]. Recent research has explored various modalities to overcome these limitations. StereoPose [43] replaces active depth with stereopsis, fusing dual-view NOCS maps through parallax attention to produce category-level 6DoF. Sodano et al. [44] designed a double-encoder network that merges RGB and depth features, which strengthens panoptic segmentation and yields more robust transparent-object poses. Keypoints that are invisible due to occlusion or truncation can be effectively inferred [45,46]. To address data scarcity, synthetic datasets have been proposed to enlarge the training corpus [47,48]. Furthermore, ClearPose [49] and TransCG [50] contributed large-scale real-world datasets, focusing on benchmarking pose estimation and depth completion for transparent objects, respectively. Zhang et al. [51] addressed the sim-to-real gap by adapting RGB-D instance segmentation to unseen objects, providing cleaner masks that benefit downstream pose estimation. Zhang et al. [52] and Lin et al. [53] enabled category-level pose estimation that handles previously unseen instances within a known category. ReFlow6D [54] learns a refractive intermediate representation for RGB-only input. Implicit-NeRF Pose [55] optimizes view-dependent radiance fields without CAD models. MVTrans [56] unifies multi-view depth, segmentation, and pose prediction in a single transformer that is trained on the photorealistic Syn-TODD dataset, while Quere et al. [57] leveraged its improved perception to achieve higher grasp success through probabilistic shared control in assistive robots.

Traditional transparent-object pose estimation methods primarily focus on estimating poses in 2D environments or simulated settings. Beyond vision, ACTOR [58] demonstrates that self-supervised tactile exploration can reconstruct category-level shapes and poses from sparse contacts, offering an alternative when transparent objects defeat optical sensors. The growing importance of 6DoF pose estimation in machine vision and industrial robotic applications has driven increased attention to this area [45].

## 3. Proposed Method

Given an RGB image and a depth image, the task of 6DoF pose estimation is to estimate a rigid transformation $p\in SE\left(3\right)$. The transformation is composed of a 3D rotation $R\in SO\left(3\right)$ and a 3D translation $t\in R^{3}$, $p=\left[R|t\right]$.

### 3.1. Overview

**Figure 1:**
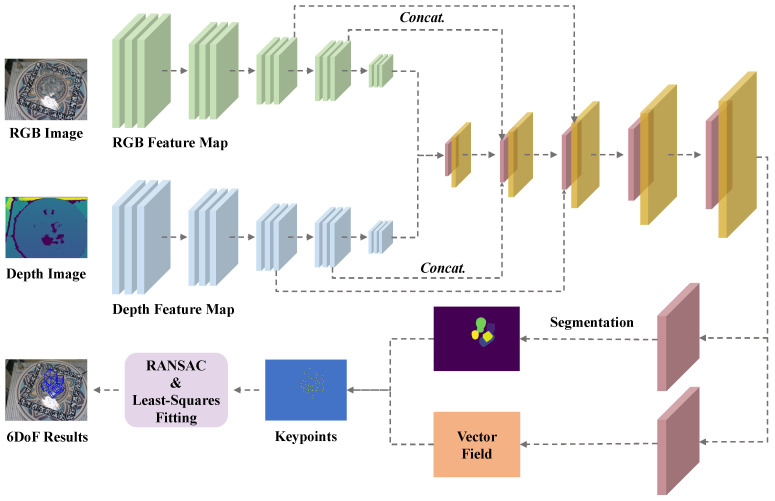
HFF6DoF network architecture. The arrows indicate the data flow. The green and blue blocks denote the RGB and depth feature maps, respectively. Yellow blocks represent the hierarchically fused features, and pink blocks indicate the decoding layers.

A hierarchical feature fusion network is proposed, as shown in Figure 1. In this network, features are extracted from the RGB and depth images by two separate ResNet34 [59] backbones, and the feature maps from the last three layers are densely fused. The fused features from the final residual layer are passed to the decoding network for up-sampling. The up-sampled features are concatenated with the densely fused features, which are then used for semantic segmentation and vector-field prediction. Finally, 6DoF poses are estimated by applying Least-Squares Fitting on the predicted and predefined keypoints.

### 3.2. Hierarchical Feature Fusion Network

The process begins with an aligned pair of images: an RGB image and a depth image. Instead of feeding unordered point clouds directly into the network, we construct a 6-channel structured geometry map. Specifically, the depth image is back-projected to align with the RGB image, forming a dense coordinate map (H×W×3). Unlike PVN3D [12] and FFB6DoF [10], which employ PointNet++ [60] to consume unordered points, our structured input allows us to utilize a convolutional neural network to efficiently extract spatially correlated geometric features.

We employ ResNet34 [59] as the backbone encoder for RGB images and structured geometry maps separately. This choice represents a strategic trade-off between efficiency and accuracy. Using deeper backbones would significantly increase computational cost and hinder real-time performance. The hierarchical feature fusion network combines the feature maps from the last three layers. Shallow layers, with a small receptive field and high resolutions, excel in capturing geometric details for smaller objects, while deep layers with a larger receptive field and lower resolutions are better suited for capturing semantic information related to larger objects.

Integrating feature maps from different scales harnesses the geometric strengths of shallow layers and the semantic capabilities of deep layers, resulting in improved 6DoF pose estimation accuracy for transparent objects.

### 3.3. Semantic Instance Segmentation

After hierarchical feature fusion, the fused features are passed through a convolutional layer to predict a semantic segmentation map, where each value represents the probability that a pixel belongs to a particular object category.

The module enables the network to extract both global and local features, with the aim of segmenting the object of interest, which is crucial for accurate keypoint localization. Moreover, the appearance and geometry information extracted for keypoint vector-field prediction further support semantic segmentation.

### 3.4. Keypoint Vector-Field Map Calculation

In this paper, we use keypoints to estimate 6DoF poses. Since the keypoint prediction network outputs a keypoint vector-field map, it is essential to calculate the accuracy of this map during training. First, we use the Farthest Point Sampling (FPS) algorithm on the object surface to define a set of keypoints. Then, we calculate the vector-field from the point cloud of the object to the set of keypoints, which serves as the ground truth.

**Keypoint selection.** Similarly to PVN3D [12] and FFB6DoF [10], we predict 3D keypoints and calculate 6DoF poses using the Least-Squares Fitting algorithm. Keypoint selection follows the FPS algorithm, as employed in PVNet [8]. Initially, the object’s center point is added to the keypoint set, and subsequent keypoints are selected iteratively based on their distance from the existing set until they reach a size of *K*.

For keypoint prediction, we calculate a vector-field map representing the vectors from each point in the point cloud to the 3D keypoints. This approach emphasizes local features, reduces background interference, and effectively handles occlusion and truncation. Even when a keypoint is not directly visible, the visible keypoints provide sufficient information to infer its position correctly.

The vector-field from the point cloud to each corresponding keypoint is calculated for each object based on semantic labels. These individual vector-fields are then combined to create a vector-field map. Notably, when two objects are occluded, the label corresponds to the foreground object. The unit vector $v_{i}\left(x\right)$ from a point *x* in the point cloud to a 3D keypoint $x_{i}^{k}$ is computed as follows:

(1) $$v_{i}\left(x\right)=\frac{x_{i}^{k}-x}{{∥x_{i}^{k}-x∥}_{2}}.$$

### 3.5. 6DoF Pose Estimation of Target Objects

**Voting-based keypoint localization.** Keypoint coordinates are obtained from the predicted keypoint vector-field through a voting process. We generate keypoint hypotheses via RANSAC. Each point in the vector-field map has a unit vector, and we randomly select two points to calculate their intersection if their unit vectors are not parallel. The connection direction from the intersection point to other points is checked against their respective unit vectors, and votes are cast for the intersection point. The point with the highest voting score is selected as the predicted keypoint.

**6DoF pose estimation.** After determining the *K* keypoints from the predicted keypoint vector-field map, we calculate the transformation matrix between the predicted keypoints ${\left\{{\tilde{x}}_{i}^{k}\right\}}_{i}^{K}$ and the keypoints on the object model ${\left\{{\bar{x}}_{i}^{k}\right\}}_{i}^{K}$. The Least-Squares Fitting algorithm minimizes the square loss to solve for the 3D rotation *R* and 3D translation *t*,

(2) $$R_{0},t_{0}=\min\sum_{i=1}^{K}{∥{\tilde{x}}_{i}^{k}-\left(R\cdot {\bar{x}}_{i}^{k}+t\right)∥}^{2}.$$

### 3.6. Loss Functions

The loss functions include both semantic segmentation and keypoint detection loss. The semantic segmentation loss ensures the network’s predicted segmentation map closely matches the ground truth semantic labels. The keypoint detection loss ensures that the predicted keypoint vector-field map is consistent with the ground truth.

First, a softmax function is used to map the predicted semantic labels to the range $\left[0,1\right]$. The semantic segmentation loss is formulated as

(3) $$L_{seg}=-\frac{1}{N}\sum_{n=1}^{N}\sum_{c=1}^{C}y_{nc}\log p_{nc},$$

where *N* represents the total number of samples, and *C* is the number of categories. If the true category of sample *n* is *c*, $y_{nc}$ equals 1; otherwise, it equals 0. $p_{nc}$ is the predicted probability that the sample *n* is classified as *c*.

Then, we use Mean Absolute Error (MAE) to compute the similarity between the predicted keypoint vector-field map and the ground truth as follows:

(4) $$L_{kpt}=\frac{1}{M}\sum_{j=1}^{M}\sum_{i=1}^{K}{∥{\tilde{v}}_{i}\left(x_{j}\right)-{\bar{v}}_{i}\left(x_{j}\right)∥}_{1},$$

where ${\tilde{v}}_{i}\left(x_{j}\right)$ is the predicted unit vector, ${\bar{v}}_{i}\left(x_{j}\right)$ is the ground truth, and *M* is the total number of points.

The final loss function is the sum of the two loss functions,

(5) $$L=L_{kpt}+\lambda L_{seg},$$

where λ is a balancing weight, empirically set to 2.

## 4. TDoF20 Dataset

**Figure 2:**
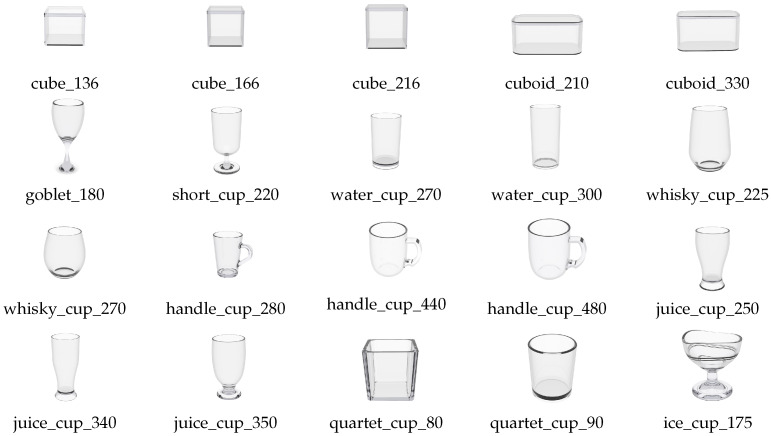
Twenty different subjects in the TDoF20 dataset.

Multiple datasets for 6DoF pose estimation have been constructed for non-transparent objects such as YCB-M [61] and HOPE [62]. However, datasets for transparent objects remain scarce. Liu et al. [63] presented a large-scale stereo image object pose estimation dataset, StereOBJ-1M, where the RGB images were the only data source. KeyPose [45], ClearPose [49], and TransCG [50] provided RGB-D datasets. These datasets were collected using stereo cameras without occlusion or truncation, with each object appearing separately in the image, which fails to reflect the complexity of real-world environments. To address this, we constructed a new dataset, TDoF20, for transparent-object 6DoF pose estimation, which is closer to practical applications. Unlike existing datasets (e.g., ClearGrasp [64] and TransCG [50]), which largely focus on depth completion tasks or simplified single-object scenarios, TDoF20 is specifically designed to address heavy inter-object occlusion and multi-object interaction in robotic grasping scenarios. By providing high-quality 6DoF pose annotations under these complex conditions, TDoF20 serves as a more challenging and realistic benchmark for robust transparent-object perception (https://github.com/MightyCrane/HFF6DoF (accessed on 26 January 2026)), see Figure 2.

### 4.1. Data Collection

**Figure 3:**
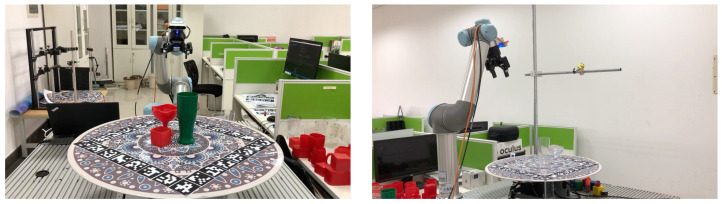
Data collection setup with UR5 robotic arm and RGB-D camera capturing objects on a turntable from multiple viewpoints.

The data collection setup is illustrated in Figure 3. The collection contains 20 single-object scenes and 20 multi-object scenes. In each multi-object scene, 3–5 objects are placed simultaneously. The data collection follows the following steps.

(i) Start the camera and UR5 robot. Next, randomly select a background sticker, and place it on the turntable, then position one or more transparent objects.

(ii) Collect RGB-D data of the transparent objects from four robot arm positions (shooting angles). For each position, the turntable rotates 400 times, at increments of 0.9°. At each increment, a pair of images (an RGB image and a depth image) is captured.

(iii) Replace the transparent object with a non-transparent object of the same shape and size. To minimize alignment errors during this manual replacement, we utilized fixed positioning slots to ensure the pose remained consistent. Then, repeat step ii to collect the images.

(iv) Repeat steps i–iii until all 20 single-object and 20 multi-object scenes are collected.

### 4.2. Data Processing

**Figure 4:**
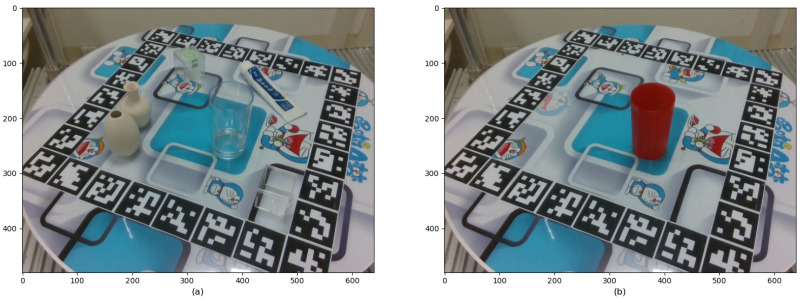
Replacement of the transparent object with its non-transparent counterpart. (**a**) The original scene containing a transparent object; (**b**) The scene with the non-transparent object used for ground truth alignment.

**Figure 5:**
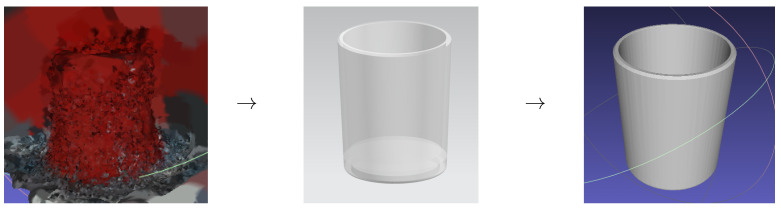
Process of building a transparent object model from the point cloud of its non-transparent counterpart. The arrows indicate the reconstruction pipeline: the raw point cloud of the non-transparent counterpart is reconstructed into a CAD model, which is then uniformly sampled to generate the final transparent object model.

**Figure 6:**
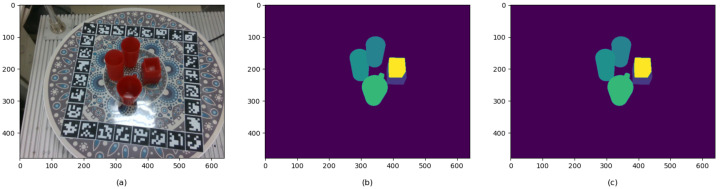
RGB image (**a**), incorrect mask (**b**), and correct mask (**c**). In the mask images (**b**,**c**), different colors represent different object instances.

After data collection, the following steps are performed to calculate the 6DoF pose and semantic labels, and create a 3D model for each object:

(i) Calculate the transformation matrix between each image and the first image using the Iterative Closest Point (ICP) algorithm applied to point clouds converted from depth images. Since depth images of transparent objects are often incomplete or corrupted, we use the depth images of the corresponding non-transparent objects.

(ii) Reconstruct the scene point cloud using the obtained transformation matrix. Since the depth images of transparent objects may be inaccurate, we rely on depth images from non-transparent objects to reconstruct the point clouds. Based on these poses, we create models of the transparent objects, see Figure 4.

(iii) Generate the transparent-object model using the point cloud obtained in step ii. Initially, we convert the point cloud into a format compatible with CAD software, SolidWorks 2019, and then apply reverse engineering for structural modeling to construct a real-sized object model. Figure 5 provides an example. To obtain the final point cloud of a transparent object, we perform uniform sampling on the model using Monte Carlo sampling in MeshLab.

(iv) Calculate the 6DoF pose and image mask for each image. As the point cloud obtained in step iii is in the camera coordinate system, it needs to be transformed into the object coordinate system to obtain the ground truth 6DoF pose. To calculate the image mask, we project the point cloud in the camera coordinate system onto a 2D plane using the camera’s internal parameters. For images containing multiple objects, a far-to-near projection order is adopted. As shown in Figure 6a, the higher water cup in the front occludes the lower one. Therefore, the lower cup is projected first, followed by the higher one.

In total, the TDoF20 dataset contains 61,886 pairs of RGB and depth images, including 31,108 single-object samples and 30,778 multi-object samples.

## 5. Experiments

### 5.1. Datasets

**Figure 7:**
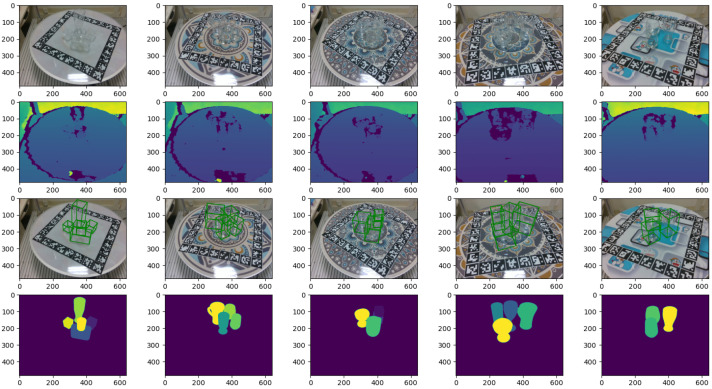
Sample data in the TDoF20-hard dataset. Row 1: RGB images; row 2: depth images (colors represent distance from the camera, where blue is closer and yellow is farther); row 3: ground-truth pose values (displayed in boxes); row 4: instance masks (different colors indicate distinct object instances). Five different background stickers were used.

We create a TDoF20-simple subdataset from all the single-object images in the TDoF20 dataset. Both our proposed method and the competitors are trained on two datasets: TDoF20-simple and TDoF20-hard. TDoF20-hard refers to the complete TDoF20 dataset. TDoF20-simple comprises a training set of 26,434 samples and a test set of 4674 samples. TDoF20-hard includes all 20 single-object scenes and 14 multi-object scenes for training, while the remaining 6 multi-object scenes are reserved for testing. The training set contains 52,747 samples, and the test set contains 9139 samples. Figure 7 shows some examples. To prevent data leakage, the TDoF20-hard test set consists of completely unseen scene sequences (Sequences 02, 08, 09, 10, 11, 15) that are physically distinct from the training configurations. Furthermore, during the inference phase, the network takes real, noisy transparent depth maps as input. The opaque depth maps described in Section 4.2 are used solely for offline ground truth generation and do not participate in network forward propagation.

### 5.2. Evaluation Metrics

We evaluate our method using two common metrics: the 2D projection metric [65] and the Average 3D Distance of model points (ADD) metric [66].

The 2D projection metric calculates the distance between the projected point sets of two 3D models given the estimated pose and the ground truth pose. The ADD metric calculates the distance between two 3D model point sets transformed by the estimated and ground truth poses. The distance is computed for symmetric objects based on the closest point distance.

### 5.3. Implementation Details

The experiments were conducted on an NVIDIA GTX 1080Ti and Ubuntu 18.04. The core algorithms were implemented using Python 3.7.12 and PyTorch 1.10.0. The proposed network includes two encoders based on ResNet34, both initialized with weights pre-trained on ImageNet. While recent works employing models such as RD3D+ [67] demonstrate the benefits of 3D CNN-based RGB-D pre-training, we opt for 2D ImageNet pre-training to prioritize inference speed. ImageNet weights are highly effective for the geometry branch, as depth maps share similar low-level visual patterns (e.g., edges and textures) with RGB images. During training, the batch size is 8, and the initial learning rate is $1\times 10^{-3}$. Following the design of PVNet, we set the number of keypoints to 9 and the weight of the final loss function to 2.

To evaluate the effectiveness of our method, we selected PVNet [8], DenseFusion [9], and FFB6DoF [10] as baselines. These methods represent state-of-the-art approaches in general RGB-D pose estimation. We excluded stereo-based methods (e.g., KeyPose [45], StereoPose [43]) from direct comparison due to the fundamental modality mismatch (Stereo Vision vs. Monocular RGB-D). Similarly, methods focusing primarily on depth completion (e.g., TransCG [50]) were not included to ensure a controlled evaluation of end-to-end pose estimation performance under standard RGB-D settings.

### 5.4. Experimental Results and Analysis

Table 1 and Table 2 show the results of the ADD and 2D projection metrics for our proposed method, HFF6DoF, compared to other methods on the TDoF20-simple and TDoF20-hard datasets. Objects with bold names are asymmetric, and the best result for each object across different methods is also highlighted in bold.

As shown in Table 1 and Table 2, the results obtained when the method was trained on TDoF20-simple are significantly higher than those obtained when it was trained on TDoF20-hard. This is primarily due to the simplicity of TDoF20-simple, which contains only single objects without occlusions or truncations. The model benefits from training on transparent objects and backgrounds already seen in the dataset. However, when faced with unseen multi-object combinations, the model’s generalization decreases, leading to a notable drop in accuracy.

**Figure 8:**
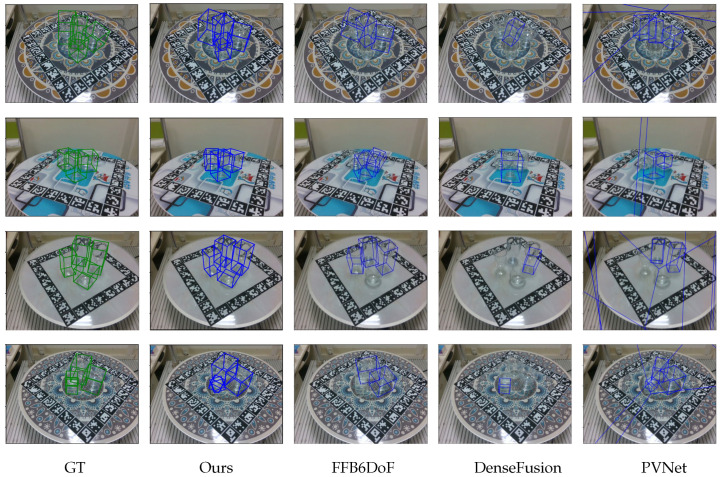
Pose estimation results of different methods on TDoF20-hard. The green 3D bounding boxes represent the Ground Truth (GT) poses, while the blue 3D bounding boxes represent the predicted poses by different methods.

Table 1 and Table 2 reveal that HFF6DoF outperforms other methods on the TDoF20 dataset. HFF6DoF achieves a 7.13% higher ADD accuracy on TDoF20-hard compared to DenseFusion, which has the highest accuracy among the other methods. Additionally, HFF6DoF achieves a 2D projection metric accuracy that is 11.91% higher than that of FFB6DoF, which also achieves the best result. This improvement is due to the hierarchical feature fusion in HFF6DoF, which leverages both RGB and depth information more effectively. Unlike cascade fusion, which mixes features layer-by-layer and tends to blur shallow geometric details in deeper layers, our approach preserves independent geometric cues through parallel branches. This is critical for transparent objects, where preserving the subtle, noisy geometric edges from the depth map is key to accurate pose estimation. In contrast, other methods like DenseFusion produce fewer fused features, and FFB6DoF overlooks certain key characteristics. PVNet, which only uses RGB images and lacks depth information, also shows lower pose estimation performance compared to HFF6DoF. The visual comparison of 6DoF pose estimation results for different methods on the TDoF20-hard dataset is shown in Figure 8.

It can also be observed that the accuracy of asymmetric objects is lower than that of symmetric objects, and small objects tend to have lower accuracy than large objects. This is because the calculation for asymmetric objects is stricter compared to that for symmetric ones, which can have more than one valid pose due to symmetry invariance. For example, a centrosymmetric object rotated by 180 degrees should have the same pose, but using a homogeneous matrix to describe the 6D pose results in different representations before and after rotation. Additionally, the ADD metric is calculated by comparing the deviations to 10% of the object’s diameter, making smaller objects more prone to being judged as having an incorrect pose. As shown in Table 1, the results obtained when the method was trained on TDoF20-hard are lower than those obtained when it was trained on TDoF20-simple. This is because in TDoF20-hard, most test samples contain multiple objects, which can occlude each other. Smaller objects may experience more occlusion, leading to reduced accuracy. This drop validates TDoF20 as a challenging benchmark designed to simulate realistic, cluttered robotic scenarios, pushing the boundaries of robust perception.

We further evaluated the efficiency of our model, as shown in Table 3. HFF6DoF achieves an inference speed of 29.38 FPS. It is noteworthy that, while our dual-stream architecture naturally increases the parameter count (56.82 M) compared to single-stream methods like DenseFusion, our method achieves the lowest computational cost in terms of FLOPs (85.12 G), which is significantly lower than that of DenseFusion (142.25 G) and FFB6DoF (215.54 G). This demonstrates that our hierarchical fusion design efficiently aggregates features without incurring excessive computational overhead, making it highly suitable for deployment on resource-constrained robotic platforms.

### 5.5. Ablation Studies

**Effect of feature fusion layers.** We evaluate HFF6DoF on TDoF20-hard using different numbers of feature fusion layers, ranging from 1 to 5. Our experiments show that the highest ADD of 50.50% is achieved with three fusion layers, after which the performance stabilizes. Fusing texture and geometric features from different scales helps the network capture both semantic and detailed information, improving performance.

**Effect of semantic segmentation modules.** We also evaluated our method with and without the semantic segmentation module. According to the experimental results, the accuracy on the ADD and 2D projection metrics is improved by 8.52% and 2.63% by using the segmentation module. The semantic segmentation module promotes 6DoF pose estimation of transparent objects based on 3D keypoint detection. It assigns a semantic label to each pixel of images and separates transparent objects from scenes, helping the keypoint detection module better locate keypoints.

### 5.6. Generalization on Non-Transparent Objects

To further validate the generalization ability of our proposed HFF6DoF and address the concern regarding evaluation on public benchmarks, we conducted additional experiments on the YCB-Video dataset [23]. YCB-Video is a widely used benchmark for 6DoF pose estimation of non-transparent objects, containing 21 objects with diverse textures and shapes. Table 4 presents the quantitative comparison results using the ADD(S) metric. Bold object names indicate symmetric objects. The “ADDS” column treats all objects as symmetric, while the “ADD(S)” column applies the standard metric (ADD for non-symmetric, ADD-S for symmetric).

As shown in Table 4, our method achieves an average ADD(S) accuracy of 91.5%, which is highly competitive compared to methods designed specifically for non-transparent objects, such as DenseFusion and PVN3D, and is comparable to FFB6DoF. Notably, HFF6DoF achieves the best performance on several specific objects, including 005_tomato_soup_can and 036_wood_block. These results demonstrate that although HFF6DoF is tailored for the challenging task of transparent-object pose estimation, its hierarchical fusion architecture is inherently robust and effective for opaque objects as well. This confirms that our method does not rely on transparency-specific priors (which would fail on opaque objects) but rather learns a generalized representation of geometry and appearance.

## 6. Conclusions

A hierarchical feature fusion network, HFF6DoF, was proposed for 6DoF pose estimation of transparent objects. The network densely fuses feature maps to make comprehensive use of RGB and depth information. A new dataset, TDoF20, was constructed, containing 61,886 pairs of RGB and depth images covering 20 types of objects in real-world scenarios. The experimental results showed that HFF6DoF outperforms state-of-the-art approaches on the TDoF20 dataset, achieving an average ADD of 50.5%. Ablation studies further demonstrated that the semantic segmentation module enhances feature extraction and overall performance. Furthermore, experiments on the YCB-Video dataset validated that HFF6DoF effectively generalizes to non-transparent objects without modification, suggesting its potential as a unified framework for mixed-material scenarios. Limitations remain regarding sensitivity to strong specular highlights that corrupt depth data. Future work will focus on addressing this limitation and exploring domain-specific pre-training to further enhance robustness in complex environments.

## Figures and Tables

**Table 1:** ADD projection values of different methods on the TDoF20-simple (S) and TDoF20-hard (H) datasets.

ADD	PVNet [8]		DenseFusion [9]		FFB6DoF [10]		HFF6DoF (Ours)	
Objects	S	H	S	H	S	H	S	H
cube_136	53.45	0.00	45.69	0.00	7.02	**0.19**	**100.00**	0.00
cube_166	56.07	0.07	78.66	3.25	7.89	0.19	**97.81**	**71.97**
cube_216	78.97	0.94	**100.00**	43.48	30.26	1.02	99.57	**62.75**
cuboid_210	72.38	0.00	99.58	**23.03**	48.68	2.32	**100.00**	12.04
cuboid_330	79.22	0.20	96.54	9.68	63.60	4.48	**100.00**	0.00
goblet_180	24.58	2.64	91.52	56.45	88.60	11.77	**100.00**	**88.47**
short_cup_220	40.42	0.26	95.00	45.45	70.61	6.52	**100.00**	**78.10**
water_cup_270	11.29	0.44	89.36	28.81	53.07	2.83	**96.71**	**64.54**
water_cup_300	25.22	0.19	**100.00**	72.67	64.91	4.68	**100.00**	**87.11**
whisky_cup_225	57.08	0.15	**100.00**	60.82	33.77	1.21	98.96	**98.67**
whisky_cup_270	62.28	2.12	**99.56**	80.33	35.96	1.24	97.99	**90.22**
**handle_cup_280**	5.00	0.00	**94.58**	**39.67**	57.46	3.09	79.41	5.25
**handle_cup_440**	12.92	0.00	84.58	13.20	75.88	7.47	**100.00**	**49.58**
**handle_cup_480**	5.83	0.19	8.06	**32.37**	58.33	3.53	**70.76**	16.24
juice_cup_250	37.24	32.67	44.35	5.07	65.79	5.38	**100.00**	**95.16**
juice_cup_340	41.25	2.94	**100.00**	**65.13**	89.91	12.75	**100.00**	53.67
juice_cup_350	24.17	0.13	41.40	28.36	78.07	7.98	**85.00**	**75.93**
quartet_cup_80	85.90	0.00	**100.00**	8.93	9.65	0.29	**100.00**	**11.94**
quartet_cup_90	77.45	0.06	93.19	17.38	7.89	0.19	**100.00**	**38.51**
**ice_cup_175**	24.12	0.00	13.33	**85.58**	52.19	2.74	**96.76**	9.92
Average	43.74	2.15	80.19	43.37	49.98	3.99	**96.15**	**50.50**

**Table 2:** 2D projection values of different methods on the TDoF20-simple (S) and TDoF20-hard (H) datasets.

2D Projection	PVNet [8]		DenseFusion [9]		FFB6DoF [10]		HFF6DoF (Ours)	
Objects	S	H	S	H	S	H	S	H
cube_136	**100.00**	0.00	37.50	0.00	**100.00**	**3.50**	**100.00**	0.00
cube_166	99.16	18.60	99.58	8.23	**100.00**	**29.83**	99.56	**77.22**
cube_216	**100.00**	12.94	**100.00**	**73.55**	**100.00**	50.19	**100.00**	64.77
cuboid_210	**100.00**	0.57	99.58	29.70	**100.00**	**40.93**	**100.00**	18.52
cuboid_330	**100.00**	2.09	90.04	29.03	**100.00**	17.10	**100.00**	**33.33**
goblet_180	84.17	59.71	92.41	3.02	94.17	81.03	**100.00**	**88.44**
short_cup_220	81.25	43.11	95.83	53.29	**100.00**	54.07	**100.00**	**80.10**
water_cup_270	74.19	31.84	94.33	34.18	75.81	49.46	**98.68**	**67.85**
water_cup_300	**100.00**	36.20	**100.00**	80.71	**100.00**	71.31	**100.00**	**93.82**
whisky_cup_225	90.42	13.27	**100.00**	67.53	99.58	78.32	98.96	**99.29**
whisky_cup_270	**100.00**	37.78	**100.00**	84.45	**100.00**	78.40	98.66	**92.18**
**handle_cup_280**	52.92	0.00	**88.75**	**42.95**	7.92	0.06	44.96	0.30
**handle_cup_440**	25.42	0.00	18.75	**1.96**	0.00	0.00	**37.08**	1.66
**handle_cup_480**	26.25	0.44	5.38	**9.12**	20.00	0.00	**46.78**	1.40
juice_cup_250	**100.00**	98.60	31.38	0.61	**100.00**	**99.71**	**100.00**	96.49
juice_cup_340	**100.00**	24.02	**100.00**	36.32	**100.00**	32.70	**100.00**	**55.54**
juice_cup_350	49.17	8.33	55.41	19.40	70.42	20.01	**85.00**	**75.93**
quartet_cup_80	**100.00**	4.21	**100.00**	20.68	**100.00**	**32.10**	**100.00**	21.98
quartet_cup_90	82.13	11.08	98.95	**50.21**	82.98	37.95	**100.00**	44.01
**ice_cup_175**	37.28	1.02	**67.14**	**88.38**	30.70	0.16	51.85	2.27
Average	80.12	20.19	78.75	36.67	79.08	38.84	**88.08**	**50.75**

**Table 3:** Computational complexity and inference speed comparison. M: million; G: giga.

Metric	PVNet [8]	DenseFusion [9]	FFB6DoF [10]	HFF6DoF (Ours)
Params (M)	36.76	**21.45**	33.85	56.82
FLOPs (G)	219.73	142.25	215.54	**85.12**
Time (ms)	102.07	**32.65**	80.77	34.04
FPS	9.80	**30.63**	12.38	29.38

Note: The best results are highlighted in bold.

**Table 4:** ADD(S) evaluation metrics of different methods on the YCB dataset. Bold values indicate the best performance.

Objects	PoseCNN [68]		DenseFusion [9]		PVN3D [12]		FFB6DoF [10]		HFF6DoF (Ours)	
	ADDS	ADD(S)	ADDS	ADD(S)	ADDS	ADD(S)	ADDS	ADD(S)	ADDS	ADD(S)
002_master_chef_can	83.9	50.2	95.3	70.7	96.0	80.5	**96.3**	80.6	96.0	**82.4**
003_cracker_box	76.9	53.1	92.5	86.9	96.1	**94.8**	**96.3**	94.6	95.9	94.0
004_sugar_box	84.2	68.4	95.1	90.8	97.4	96.3	**97.6**	**96.6**	**97.6**	96.4
005_tomato_soup_can	81.0	66.2	93.8	84.7	96.2	88.5	95.6	89.6	**96.5**	**90.9**
006_mustard_bottle	90.4	81.0	95.8	90.9	97.5	96.2	**97.8**	**97.0**	97.7	96.8
007_tuna_fish_can	88.0	70.7	95.7	79.6	96.0	89.3	96.8	88.9	**97.0**	**90.6**
008_pudding_can	79.1	62.7	94.3	89.3	**97.1**	**95.7**	**97.1**	94.6	95.0	89.1
009_gelatin_box	87.2	75.2	97.2	95.8	97.7	96.1	**98.1**	**96.9**	97.4	96.5
010_potted_meat_can	78.5	59.5	89.3	79.6	93.3	**88.6**	**94.7**	88.1	92.5	84.0
011_banana	86.0	72.3	90.0	76.7	96.6	93.7	97.2	**94.9**	**97.6**	93.9
019_pitcher_base	77.0	53.3	93.6	87.1	97.4	96.5	**97.6**	**96.9**	97.1	96.1
021_bleach_cleanser	71.6	50.3	94.4	87.5	96.0	93.2	**96.8**	**94.8**	96.3	92.2
**024_bowl**	69.6	69.6	86.0	86.0	90.2	90.2	**96.3**	**96.3**	84.1	84.1
025_mug	78.2	58.5	95.3	83.8	**97.6**	**95.4**	97.3	94.2	96.1	91.1
035_power_drill	72.7	55.3	92.1	83.7	96.7	95.1	97.2	95.9	**97.3**	**96.3**
**036_wood_block**	64.3	64.3	89.5	89.5	90.4	90.4	92.6	92.6	**92.8**	**92.8**
037_scissors	56.9	35.8	90.1	77.4	96.7	92.7	**97.7**	**95.7**	94.4	85.9
040_large_marker	71.7	58.3	95.1	89.1	**96.7**	**91.8**	96.6	89.1	94.4	85.7
**051_large_clamp**	50.2	50.2	71.5	71.5	93.6	93.6	**96.8**	**96.8**	94.5	94.5
**052_extra_large_clamp**	44.1	44.1	70.2	70.2	88.4	88.4	**96.0**	**96.0**	94.5	94.5
**061_foam_brick**	88.0	88.0	92.2	92.2	96.8	96.8	**97.3**	**97.3**	96.3	96.3
Average	75.8	59.9	91.2	82.9	95.5	91.8	**96.6**	**92.7**	95.7	91.5

## Data Availability

The TDoF20 dataset is publicly available at https://github.com/MightyCrane/HFF6DoF (accessed on 26 January 2026).
